# On the nature of extraversion: variation in conditioned contextual activation of dopamine-facilitated affective, cognitive, and motor processes

**DOI:** 10.3389/fnhum.2013.00288

**Published:** 2013-06-13

**Authors:** Richard A. Depue, Yu Fu

**Affiliations:** Human Development, Cornell UniversityIthaca, NY, USA

**Keywords:** dopamine, extraversion, conditioning, cognition, motor velocity, positive affect

## Abstract

Research supports an association between extraversion and dopamine (DA) functioning. DA facilitates incentive motivation and the conditioning and incentive encoding of contexts that predict reward. Therefore, we assessed whether extraversion is related to the efficacy of acquiring conditioned contextual facilitation of three processes that are dependent on DA: motor velocity, positive affect, and visuospatial working memory. We exposed high and low extraverts to three days of association of drug reward (methylphenidate, MP) with a particular laboratory context (Paired group), a test day of conditioning, and three days of extinction in the same laboratory. A Placebo group and an Unpaired group (that had MP in a different laboratory context) served as controls. Conditioned contextual facilitation was assessed by (i) presenting video clips that varied in their pairing with drug and laboratory context and in inherent incentive value, and (ii) measuring increases from day 1 to Test day on the three processes above. Results showed acquisition of conditioned contextual facilitation across all measures to video clips that had been paired with drug and laboratory context in the Paired high extraverts, but no conditioning in the Paired low extraverts (nor in either of the control groups). Increases in the Paired high extraverts were correlated across the three measures. Also, conditioned facilitation was evident on the first day of extinction in Paired high extraverts, despite the absence of the unconditioned effects of MP. By the last day of extinction, responding returned to day 1 levels. The findings suggest that extraversion is associated with variation in the acquisition of contexts that predict reward. Over time, this variation may lead to differences in the breadth of networks of conditioned contexts. Thus, individual differences in extraversion may be *maintained* by activation of differentially encoded central representations of incentive contexts that predict reward.

## Introduction

Extraversion represents a higher-order personality trait that has been identified in virtually all classificatory systems of the structure of personality, including Eysenck and Gray's models (Gray, 1994), the Five-Factor model (Costa and McCrae, 1992), Tellegen's Multidimensional Personality Questionnaire (MPQ) model (Tellegen and Waller, 2008), and Zuckerman's Alternative Five-Factor model (Zuckerman, 2002). The phenomenology of extraversion is described similarly in all of these models, and is characterized by adjectives that connote a state of positive affect and strong motivation of desire and wanting, as well as by feelings of being excited, enthusiastic, active, peppy, strong, confident, and optimistic (Watson and Tellegen, 1985; Berridge, 2004).

Jung (1921) insightfully placed this positive motivational state in a larger context in his description of extraversion. He suggested that extraversion is characterized by broad engagement with the environment which is supported by the positive affective state emphasized by others. Jung's notion suggests that there is a broad class of environmental stimulus that elicits positive affective engagement, and Gray (1994) extended that notion by arguing that the stimulus class is composed of rewards. Thus, extraversion may represent individual differences in the extent to which environmental rewards elicit positive affective engagement as a means of obtaining those rewards.

Due to conceptually similar phenomenological features, we drew an analogy between this positive affective state in humans and incentive motivation as described in the animal literature (Depue and Collins, 1999; Depue and Morrone-Strupinsky, 2005; Depue and Fu, 2012). Incentive represents a motivational system identified in all mammals, and is elicited by the broad stimulus class of unconditioned and conditioned incentive stimuli that induce forward locomotion and strong subjective feelings of reward. This analogy suggested that, if extraversion represents the manifestation of an incentive reward system, then the trait may be in part influenced, as this motivation is in animals, by the activity of the mesocorticolimbic dopamine (DA) projection system. This projection system originates mainly in the ventral tegmental area (VTA) of the midbrain, and sends afferents to several limbic regions, including the nucleus accumbens (NAc) in the ventral striatum and the amygdala, and to many cortical regions, including the orbital cortex (Depue and Collins, 1999; Depue and Morrone-Strupinsky, 2005; Fields et al., 2007).

In rats and monkeys, dose-dependent DA receptor activation in the VTA-NAc pathway mediates the acute rewarding effects of stimulants, and facilitates a broad array of incentive motivated behaviors, including locomotor activity to novelty and food; as well as exploratory, aggressive, affiliative, and sexual behavior (Depue and Collins, 1999; Berridge, 2007). In single-unit recording studies in monkeys, large populations of VTA DA neurons are activated preferentially by appetitive incentive stimuli (Schultz et al., 1995, 1997; Mirenowicz and Schultz, 1996; D'Ardenne et al., 2008; Schroeder et al., 2008), and DA cells, most numerously in the VTA, respond in proportion to the magnitude of both conditioned and unconditioned incentive stimuli (Fields et al., 2007; Schultz, 2007; Bromberg-Martin et al., 2010). Similarly, NAc cells increase firing to primary and conditioned signals of reward and novelty during intervals when reward is expected, and during engagement in rewarding social activity.

In humans, incentive motivation is associated with both positive *emotional* feelings such as elation and euphoria, and *motivational* feelings of desire, wanting, craving, potency, and self-efficacy (Depue and Collins, 1999). This is in contrast to positive feelings that accompany reward consummation, which is associated with feelings of gratification, quiescence, liking, and calm pleasure (Depue and Morrone-Strupinsky, 2005; Smillie et al., 2012). DA activity is related to the former, but not the latter, subjective emotions. Thus, neuroimaging studies have found that, during acute cocaine or amphetamine administration, the intensity of a participant's subjective euphoria increased in a dose-dependent manner in proportion to DA-agonist binding to the DA uptake transporter (and hence DA levels) in the ventral striatum (Volkow et al., 1997). Moreover, DA-induced activity in the NAc was linked equally strongly (if not more strongly) to motivational feelings of desire, wanting, and craving, as to the emotional experience of euphoria (Breiter et al., 1997). And the degree of activation by positive or rewarding stimuli or agonist-induced DA release in healthy human ventral striatum and other regions of reward circuitry (e.g., amygdala, medial orbitofrontal cortex, and anterior cingulate cortex) assessed by fMRI and PET were correlated strongly with (i) feelings of euphoria, (ii) extraversion and similar traits of novelty seeking and affective impulsivity, (iii) DA-relevant gene polymorphisms, and (iv) pharmacological indicators of DA functioning (Depue et al., 1994; Depue, 1995; Berke and Hyman, 2000; Drevets, 2001; Canli et al., 2002; Kumari et al., 2004; Knutson and Cooper, 2005; Mobbs et al., 2005; Reuter and Hennig, 2005; Reuter et al., 2006; Deckersbach et al., 2006; D'Ardenne et al., 2008; Zald et al., 2008; Smillie et al., 2009; Bromberg-Martin et al., 2010; Buckholtz et al., 2010; Haber and Knutson, 2010; Baik et al., 2012). Hence, taken together, the animal and human evidence supports the notion that the VTA DA-NAc pathway is a primary neural circuit for incentive reward (Bromberg-Martin et al., 2010; Haber and Knutson, 2010; Sesack and Grace, 2010), and that extraversion is related to activity in that pathway (Wacker et al., 2006, 2012, 2013).

**Figure 1:**
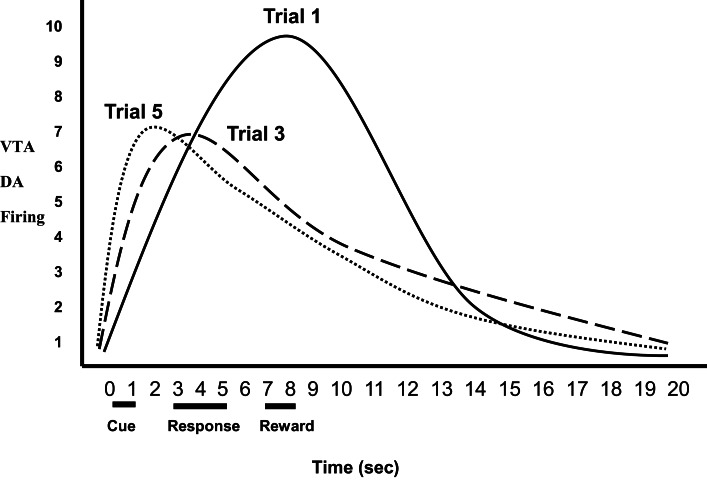
**Relative ventral tegmental area (VTA) dopamine (DA) firing as a function of trial**. VTA DA neurons show increased activity in the presence of neutral stimuli that consistently predict reward, and a concurrent decrease in activity to the unconditioned rewards, until DA responding has transferred completely to the conditioned incentive stimuli (Trials 1–5).

While VTA DA activation is critical for inducing incentive motivation in NAc, VTA DA neuron responses also play a role in facilitating the association between those stimuli that predict reward (i.e., conditioned stimuli) and motivated behavior that obtains reward (Schultz et al., 1997; Montague et al., 2004; Schultz, 2007). With regard to associative learning, mere DA neuron activation without exogenous reward produced a preference for the context paired with phasic DA firing. Concordantly, DA neuron firing was gradually time-locked to the presentation of a conditioned cue that predicted sucrose delivery, and phasic DA release correlated positively with conditioned approach behavior toward the cue (Stuber et al., 2008). This associative process includes the following steps. The optimal stimuli for activating VTA DA neurons are *unpredicted* unconditioned rewards (e.g., food, sweet liquid). Such biologically salient stimuli are evaluated for their emotional significance in the basolateral amygdala (BLA) and medial orbital frontal cortex (mOFC). If such stimuli have sufficient incentive salience, these and other corticolimbic areas then activate VTA DA neurons (Berke and Hyman, 2000; Myer-Lindenberg et al., 2005; Fields et al., 2007; Kauer and Malenka, 2007; Stuber et al., 2008; Zellner and Ranaldi, 2010), which release DA into the NAc as a means of increasing incentive motivation to obtain the reward. Subsequently, neutral cues in the current context that consistently predict reward are associated with reward (become CSs) in the BLA and mOFC (Elliott et al., 2003; Gottfried et al., 2003; Simmons et al., 2007; D'Ardenne et al., 2008), which in turn activate VTA DA neurons prior to the occurrence of primary reward (Zellner and Ranaldi, 2010). This process is shown in Figure 1 during an experiment's progression: VTA DA neurons show increased activity in the presence of neutral stimuli that consistently predict reward, and a concurrent decrease in activity to the unconditioned reward, until DA responding has transferred completely to the conditioned incentive stimuli (Schultz et al., 1997; Galvan et al., 2005; Day et al., 2007; Schultz, 2007; Stuber et al., 2008). Thus, VTA DA discharge ratchets backward in time so as to respond to earlier and earlier predictors of reward. Therefore, DA activity is critical to the control of appetitive behavior by conditioned incentive stimuli—specifically, to link stimuli predicting reward, which activate VTA neurons, to the response-facilitation mechanism in the NAc (Schultz et al., 1997; Depue and Collins, 1999; Nestler, 2001; Depue and Morrone-Strupinsky, 2005; Berridge, 2007; Stuber et al., 2008; Zellner and Ranaldi, 2010).

The acquisition of a reward-predictive neural structure is enhanced when VTA DA activation results in release of DA in the NAc. DA release in the NAc plays a critical role in the formation of complex contextual ensembles that predict the occurrence of reward in a much more detailed manner than do single CS incentives (Depue and Morrone-Strupinsky, 2005; Depue and Fu, 2012). The array of stimuli that comprise the full context that precedes the occurrence of primary reward converge on the NAc (O'Donnell, 1999). These corticolimbic inputs originate from many perceptual processing pathways, but importantly also from those areas that compute the incentive salience of contextual stimuli, including the BLA, mOFC, and extended amygdala (e.g., bed nucleus of the stria terminalis) (Groenewegen et al., 1999a,b; O'Donnell, 1999; Berke and Hyman, 2000; Depue and Morrone-Strupinsky, 2005). The end product of this compression is a contextual ensemble that is encoded for incentive salience or value. That ensemble is further compressed in a cortico-cortical loop, which terminates in the mOFC where the ensemble is associated with an expected outcome (i.e., probability and magnitude of reward; Alexander et al., 1990; O'Donnell, 1999; Amodio and Frith, 2006). It is not surprising then that it is the mOFC that provides the major source of activation of VTA DA neurons when predictive contexts of reward occur (Taber et al., 1995; Carr and Sesack, 2000; Zellner and Ranaldi, 2010). The magnitude of the encoded incentive salience of the mOFC contextual ensemble is thus translated into the magnitude of mOFC-VTA DA activation and, in turn, NAc DA-facilitated incentive motivation.

The acquisition of contextual ensembles is strongly dependent on DA in the NAc. Corticolimbic regions carrying contextual information innervate NAc neurons in close proximity to VTA DA projections to the NAc (O'Donnell, 1999; Depue and Morrone-Strupinsky, 2005; Sesack and Grace, 2010). It is here that DA facilitates the development of long-term potentiated connections of corticolimbic afferents to NAc neurons (Nestler, 2001; Goto and Grace, 2005; Kauer and Malenka, 2007; Shen et al., 2008; Stuber et al., 2008). Presumably, the more DA that is released in the NAc, (a) the greater the strengthening of the connection of contextual afferents on NAc neurons, and (b) the greater the number of afferents thus facilitated. *Hence, variation in DA input to the NAc will modulate the strength of the contextual ensemble, and hence the capacity of that ensemble to subsequently elicit incentive motivation, positive affect, and approach behavior (i.e., extraverted behavior)*.

The importance of this model is that individual differences in VTA DA-NAc reactivity to reward, as found in extraversion, could modify the associative conditioning of unconditioned rewards to neutral contextual cues, and thereby create differences in the strength and breadth of individuals' networks of reward-relevant contexts. Exactly this prediction has been confirmed in animal studies, where a significant correlation between DA functioning and contextual conditioning was demonstrated (Hooks et al., 1992; Cabib, 1993; Jodogne et al., 1994; Wassum et al., 2011). The implication of these findings is that variation in the strength and breadth of reward-predictive contextual networks could play a critical role in the *maintenance* of individual differences in extraverted behavior over time.

Expanding a small preliminary study on conditioning and extraversion, we more fully investigated these possibilities by studying the *acquisition* and *extinction* over seven consecutive days of conditioned contextual facilitation of DA-modulated motor, affective, and cognitive processes in a DA agonist (methylphenidate)-paired context in high and low subgroups of extraverts. We predicted and found that high extraverts who had context paired with methylphenidate showed significantly greater conditioned contextual facilitation across all three processes relative to low extraverts. Indeed, low extraverts showed little, if any, conditioning under these experimental conditions. Moreover, conditioning was verified not only on a conditioning Test day, but also by demonstrating (a) robust conditioned responses on the first day of extinction under placebo in the absence of unconditioned drug effects, and (b) the decay of conditioned responding over a three-day extinction period.

## Materials and methods

### Design

**Figure 2:**
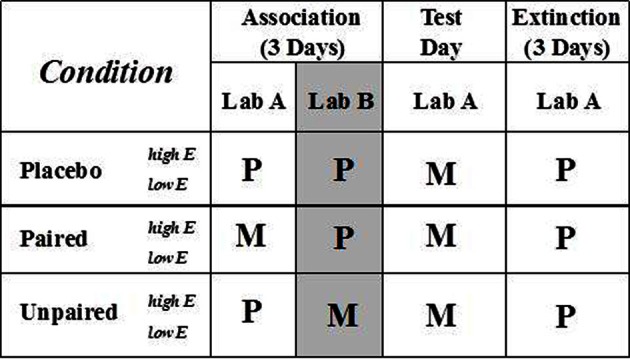
**Study design and experimental conditions**. See text for details. M, methylphenidate; P, placebo.

A study design with three consecutive phases was used (Figure 2): (i) *Association* (days 1–3), in which MP or placebo (lactose) is associated with laboratory context for three days. MP and placebo were administered in identical capsules double-blind to drug and extraversion score. On the basis of preliminary studies, three Association days were used; even one day with low doses of DA agonist is adequate in rats to demonstrate acquisition of contextual association to incentive processes (Anagnostaras and Robinson, 1996; Robinson and Berridge, 2000); (ii) *Test* (day 4), in which degree of contextual facilitation of responding is assessed under MP conditions; and (iii) *Extinction* (days 5–7), three days of placebo, where the first extinction day (day 5) assessed the presence of conditioned context-facilitated responding in the absence of unconditioned drug effects, which provides direct evidence of a motivational effect of conditioned cues (Anagnostaras and Robinson, 1996; Everitt et al., 2001).

Three experimental conditions, each with high and low subgroups of extraverts (i.e., six groups total), paired MP exposure with laboratory context (*Paired*) or did not (*Unpaired* and *Placebo*). On each Association day, all three experimental conditions received MP or placebo in each of two contextually distinct laboratories (Lab *A*, followed by Lab *B*—in which participants read emotionally neutral magazines supplied by the experimenter, as they also did in Lab A when not involved in tasks). This procedure equated Paired and Unpaired conditions for MP exposure but within different laboratory contexts (see Figure 2) (Anagnostaras and Robinson, 1996). Following previous research (Anagnostaras and Robinson, 1996; Robinson and Berridge, 2000), the context of Labs A and B differed in physical dimensions, flooring, wall colors and decorations, lighting, furniture, and experimenters. Because psychostimulants, including MP, strongly *amplify* conditioned-cue activation of behavior via DA release in the NAc (Parkinson et al., 1999; Robinson and Berridge, 2000; Everitt et al., 2001), all conditions received MP on Test day. MP was administered on Test day, because expression of conditioned drug effects are context-dependent. Therefore, despite receiving MP, the control groups above should not express facilitation of responding as should the group that has acquired conditioned facilitation. This allowed an assessment on Test day of the extent to which contextual cues had acquired incentive properties in the Paired condition relative to unconditioned effects of MP in Unpaired and Placebo groups.

### Participants

The MPQ (Tellegen and Waller, 2008) extraversion scale was used. It correlates with EPQ extraversion (0.62, *P* < 0.01), incorporates content of the extraversion scales measured by the NEO-PI (Costa and McCrae, 1992; Church, 1994), is influenced by strong genetic variation (Tellegen et al., 1988), and its positive affect or emotionality interpretation is supported by convergent-discriminant relations to the state dimension of positive affect (Zevon and Tellegen, 1982; Watson and Tellegen, 1985; Tellegen and Waller, 2008). MPQ extraversion scores were obtained from 92% (*N* = 2997) of Cornell freshmen, which has an MPQ profile equivalent to other university samples and to the general population within the age range of 19–24 years (Tellegen and Waller, 2008). High and low extraversion subgroups were randomly selected from the top and bottom deciles, respectively, of MPQ extraversion scores, and then were randomly assigned to the three experimental conditions. Selected participants were medically and psychiatrically normal and taking no medications, as verified blind to MPQ score by (i) medical interview and physical exam by a physician, and (ii) psychiatric interview using the latest version of the SCID (non-patient version), DSM-IV criteria, and the Personality Disorders Examination (Loranger, 1994) for Axis II disorders. We excluded participants with (a) cardiovascular, immune, or endocrine disorders or who were taking medications for these or other conditions that might interact with MP; (b) Axis I and II disorders because such conditions may affect DA functioning in unpredictable ways; (c) substance abuse or dependence; and (d) a recent (within last two years) smoking history, since nicotine may interact with DA. We have found that frequency of smokers does not differ above or below the MPQ extraversion median. To detect illicit drug use, participants received a confidential drug screen the day prior to each study day. No illicit drug use was detected.

Of the 74 initially selected male participants, 70 (95%) participated. As is expected due to strict decile selection criteria, MPQ extraversion scores did not differ significantly between comparisons of all low subgroup combinations (all *P*'s <0.70) nor between comparisons of all high subgroup combinations (all *P*'s < 0.70) across experimental conditions (Table 1). The 70 participants were also selected on the basis of their falling within the middle six deciles on MPQ Negative Emotionality (Neuroticism) and Constraint (impulsivity scale). Therefore, high and low extraversion participants were equivalent (not significantly different) on these other MPQ traits. Males (Caucasian; age: 19–21 years; weight: 62–88 kg) rather than females were used because DA efficacy markedly varies across the menstrual cycle (Depue et al., 1994). The number in each of the six experimental groups is: Paired High Extraversion: (PH = 15); Paired Low Extraversion: (PL = 15); Unpaired High Extraversion: (UPH = 10); Unpaired Low Extraversion: (UPL = 10); Placebo High Extraversion: (PBH = 10); Placebo Low Extraversion: (PBL = 10). Because the critical comparison in this study is between paired high vs. paired low extraversion, the *N* for those two groups is higher than for the other groups. Written informed consent was obtained from all participants in a protocol approved by Cornell University's institutional review board.

**Table 1:** **MPQ Extraversion scores for low and high extraversion subgroups in each condition**.

**Condition**	**Mean (SD)**
**PLACEBO**	
Low (*n* = 10)	5.71 (1.69)
High (*n* = 10)	33.65 (2.60)
**UNPAIRED**	
Low (*n* = 10)	5.86 (1.51)
High (*n* = 10)	33.42 (2.26)
**PAIRED**	
Low (*n* = 15)	5.79 (1.43)
High (*n* = 15)	33.49 (1.73)

### Methylphenidate (MP)

MP was used because (a) MP exerts its DA-agonist effects by increasing release of DA from presynaptic terminals, thereby activating an array of DA receptor subtypes; (b) MP binds with similar or greater magnitude to the same DA-uptake transporter as cocaine and amphetamine at presynaptic sites in cortex and striatum, especially the NAc; (c) regional distribution of MP binding in human brain is almost identical to cocaine; and (d) MP strongly induces NAc-facilitated incentive motivated behaviors, including (i) rewarding properties in conditioned place preference, (ii) self-administration in primates, and (iii) positive affect, energy, and euphoria in humans at doses of 0.5 mg/kg or less that correlates with its % DA-uptake binding in ventral striatum (Volkow et al., 1995, 1997, 1998, 2001).

MP was also used because of its specificity of action to DA at doses used here. In individual limbic and coritical brain regions, there are varying mixtures of D1, D2, D3, D4, and D5 receptors (Strange, 1993). The control of motor, emotion, and motivation processes by DA in these brain regions will, therefore, be dependent on DA interacting with various combinations of receptor isoforms. With respect to behavioral effects of D1 and D2 and D1/D2 mixed agonists and antagonists in interaction with MP, MP has its behavioral effects via both D1 and D2 receptors in a dose-dependent manner (Koek and Colpaert, 1993; Strange, 1993). Importantly, compounds not directly involving DA receptors, and compounds with antagonist properties at CNS receptors other than DA (including alpha 1 and 2 and beta noradrenergic, and 5HT 2 and 1A receptor antagonists), either did not interact with MP behavioral effects, or did so only at such high doses that extreme behavioral adverse effects occurred (Koek and Colpaert, 1993). Moreover, affinitiy for the 5HT transporter is not only much lower for MP than amphetamine and cocaine, but also affinity for this transporter is not associated with the reinforcing properties of MP (Ritz et al., 1987; Little et al., 1993). Thus, at the relatively low dose used in the current study, MP's major effects appear to be on both D1 and D2 (and perhaps other DA) receptor families. Since DA facilitation of incentive motivation, positive affect, and initiation of locomotion appears to involve at least both D1 and D2 receptors (Depue and Collins, 1999), MP is a better agonist to study extraversion processes than bromocriptine or bupropion (Vassout et al., 1993), which both have mainly D2 receptor effects. MP also appears to have a more specific DA transporter binding affinity, relative to noradrenergic and serotonergic affinities (Weiner, 1972), than amphetamine and to some extent cocaine.

Percent binding of MP to the DA-uptake transporter provides one means of judging the “saturation” effects of an MP dose, and is correlated significantly with induced positive affect in humans (Volkow et al., 1997). We used an oral MP dose of 0.6 mg/kg based on the fact that at this dose (a) % DA transporter binding is ~80% or more (Volkow et al., 1998, 2001); (b) a sufficiently long, stable peak plateau (~90 min) is associated with the positive affect effects of MP (Volkow et al., 1997, 1998), permitting sufficient time for our task administration (~1 h) at peak MP concentrations; (c) no significant negative affect is observed; and (d) clearance is ~10 h, indicating wash-out by the next day (Volkow et al., 2001). In addition, in humans, retest stability for the binding and time-course characteristics of MP (0.5 mg/kg) is very high (Volkow et al., 1995). Finally, in humans, MP has a very low adverse effect profile when orally administered acutely in low dose (0.5 mg/kg or less) (Aoyama, 1994; Wang et al., 1994; Volkow et al., 1995).

### Experimental stimuli

The extent to which MP-induced reward is associated with context in the Paired condition is reflected in facilitation of responding elicited by *general contextual features* of Lab A. General context-reward association, like conditioned place preference, is an implicit Pavlovian process that is acquired more readily and with greater resistance to extinction than is the pairing of explicit, discrete stimuli with reward (Holland, 1992; Graybiel, 1998). The number of conditioning sessions required for general context vs discrete stimuli in animals is ~1:20 session ratio, respectively. To assess the success of associative conditioning of Lab A to MP, we used five 20-s video clips that differed in their (i) association with laboratory context, (ii) MP drug effects, and (iii) inherent incentive value. The five video clips were presented in Lab A via VCR in randomized order, each separated by a 1-min rest interval, on a 56-inch TV monitor located 12 feet in front of participants.

The content of *three* of the video clips, shown on Association day 1 and Test day 4, were initially incentive-neutral, but differed in their representation of the Lab A context and in their association with MP drug reward: (i) *Library*: a moving pan across the front of Cornell's main library, which has no association with Lab A or drug reward; (ii) *Labfront*: a moving video pan across the front of Lab A, which participants continually faced during the study because they were seated facing the front of the lab; and (iii) *Portrait*: a large poster of a female portrait in the front of Lab A. The latter two stimuli vary in two other ways: *Labfront* (i) represents an implicit general contextual stimulus, which is rapidly and strongly conditioned in animals, and (ii) such general contextual stimuli are likely processed in the dorsal visual stream (i.e., via peripheral vision). In contrast, *Portrait* (i) represents an explicit, discrete stimulus object that is conditioned more slowly in animals and (ii) such discrete stimuli are likely processed in the ventral visual stream (i.e., as object recognition). Differential facilitated responding on Test day 4 is a direct test of an acquired incentive salience for *Labfront* and *Portrait* compared to *Library*.

*Two* additional previously validated video clips (Morrone et al., 2000; Morrone-Strupinsky and Depue, 2004), also shown on Association day 1 and Test day 4, had no association with drug reward or the general context of Lab A (outside of the 5-minute exposure on day 1). The two clips, however, differed in inherent incentive value and appetitive approach motivation, to which extraverts respond vigorously, but not in calm pleasurable feelings, to which extraverts do not respond vigorously (Morrone et al., 2000; Morrone-Strupinsky and Depue, 2004; Smillie et al., 2012): (iv) *Rainforest (low incentive)*: neutral rainforest scenes, and (v) *Football (high incentive and approach motivation, rather than a calm pleasurable emotional state*: a triumphant football game sequence (scoring of a touchdown). The rationale for comparing these two clips is to assess whether the Lab A context had acquired facilitatory effects on unfamiliar stimuli that had not been paired with Lab A or with MP. The incentive response elicited by any stimulus is a joint function of both the conditioned incentive value of the context and of the inherent incentive value of the unfamiliar stimulus (Jodogne et al., 1994; Schultz et al., 1997; Robinson and Berridge, 2000). Stimuli with little inherent incentive value, like *Rainforest*, will not be facilitated substantially by a conditioned context. While the incentive response to the *Football* clip relative to the *Rainforest* clip is expected to naturally differ on day 1, whether that incentive response will evidence an enhancement on day 4 relative to day 1 depends on the success of the conditioning procedure in interaction with the natural incentive value of the unfamiliar stimulus. Therefore, if there is an enhanced incentive response to *Football* on day 4 relative to day 1, but no enhancement for *Rainforest*, then one may conclude that the enhanced response to *Football* on day 4 was dependent on contextual conditioning (Robinson and Berridge, 2000).

Preliminary research showed that *Library, Rainforest, Labfront*, and *Portrait* were initially rated on both the 10-point positive and negative affect scales used in this study (see below) as neutral in affective state [*N* = 50 college males; Positve Affect Means (SDs) = 1.1 (0.05), 1.01 (0.03), 1.08 (0.04), 2.03 (0.07), respectively, where a rating of 1 or 2 = neutral affect state]. *Football* was rated 4.1 (1.2), where 4 = mild positive affect state. Mean negative affect ratings were generally around 1, and did not exceed 2.2 (neutral affect state).

### Measures

Three variables, measured only in Lab A, indexed conditioned context facilitation on motor, affective, and working memory processes. All three variables are strongly dependent on VTA DA projections to the NAc or dorsolateral prefrontal cortex (working memory variable). The three variables were assessed only on Association day 1 and on Test day 4 to avoid excessive task repetition, with affective and motor variables being measured (in that order) after each of the video clips. Working memory was measured only once on these two days, immediately after the video clip presentations. During the Extinction phase, only motor and affective responses to video clips were measured—on the first (day 5) and final (day 7) days of extinction. The cognitive task was not assessed in Extinction, because it is subject to repetition effects (Luciana et al., 1992).

#### Motor velocity

*Velocity* of motor behavior is (i) specifically related to incentive processes facilitated by DA predominantly in the NAc (Le Moal and Simon, 1991; Depue and Collins, 1999), (ii) activated by drug-associated conditioned cues (Hyman and Malenka, 2001), and (iii) correlates (*r* = 0.68, *P* < 0.01) with % DA-uptake binding in human NAc (Volkow et al., 1998). Therefore, velocity of finger tapping was measured as in Volkow et al. (1998). Finger tapping was performed on a laptop computer space bar for 6 s using the dominant hand with palm resting on the laptop base so that taps were performed solely by finger-wrist movement. To control for variation in reaction time (RT), which affects number of taps in the first second, only the last 5 s of tapping were analyzed. Preliminary studies using 20 s of tapping showed that differences between individuals are most marked in the initial 5-s period of tapping (after 1 s correction for RTs).

#### Positive affect

Positive affect, which reflects a state of positive incentive motivation (Zevon and Tellegen, 1982; Watson and Tellegen, 1985; Watson and Clark, 1997; Depue and Collins, 1999; Tellegen and Waller, 2008), was assessed by a rating scale similar to a previously validated scale described in detail elsewhere (Morrone et al., 2000; Morrone-Strupinsky and Depue, 2004). This and similar scales have excellent internal consistencies, retest reliabilities, and factor homogeneity (Watson and Tellegen, 1985; Watson et al., 1988; Krauss et al., 1992). They are also correlated with (i) % DA-uptake binding specifically in human ventral striatum (Volkow et al., 1997), (ii) DA-agonist challenge and responses to the video material used here (*r* = 0.57, *P* < 0.01) (Depue et al., 1994; Volkow et al., 1997; Morrone et al., 2000; Morrone-Strupinsky and Depue, 2004), and (iii) extraversion (*r* = 0.49, *P* < 0.01) (Morrone et al., 2000). Intraclass correlation between MP-induced peak affect ratings obtained 2–3 months apart is 0.58 (*P* < 0.05; *N* = 20, ranging from top to bottom decile on MPQ extraversion). Negative affect state was also rated at the same times as positive affect, but the former showed little (non-significant) variation from 1 to 2 (neutral mood state), and no significant activation by MP. Therefore, negative ratings are not discussed further.

The positive and negative affect rating scales are visual analog scales ranging from 1 (neutral affect state) to 10. Point 10 was anchored by adjectives found to be most highly correlated with positive and negative affect states (Watson and Tellegen, 1985). The positive adjective anchors were: *active, elated, enthusiastic, excited, peppy, strong* (where all adjectives were listed under point 10 on the scale). Participants were instructed to rate their emotional response on the scale to each clip.

The positive affect rating scale was displayed on a laptop monitor, and ratings were made directly on computer. For the affect and motor measures, the stimulus–response sequence was: (a) audiovisual prompt on the monitor, preparing the participant for the video clip, (b) video clip, (c) positive affect rating (~3 s), (d) 6 s of tapping, the timing of which started with the first tap and ended with an audio stop-beep produced by the laptop, and (e) 1-min rest interval between video clips. Participants were trained *prior* to the study on the laptop, tapping procedure, and rating scales.

#### Visuospatial working memory task

This measure reflected conditioned incentive effects derived from the general laboratory context of Lab A. The task, validated and described previously (Luciana et al., 1992, 1998; Luciana and Collins, 1997), is dependent in primates and humans on VTA DA projections to dorsolateral prefrontal cortex, and is facilitated by MP (Oades and Halliday, 1987; Luciana et al., 1992, 1998; Luciana and Collins, 1997; Devilbiss and Berridge, 2008; McNab et al., 2009; Aart et al., 2011). Briefly, during each trial, participants observed a central fixation point (a black “+”) on a computer monitor for 3 s. Next, a visual cue (a blackened circle against a white background) appeared in peripheral vision within a 360° Circumference for 200 ms (too brief to make a saccadic eye movement), after which the cue and fixation point disappeared and the screen blackened for delay intervals of 0.5 s, 4.0 s, or 8.0 s. After the delay, participants indicated the screen location of the cue with a light pen (FTG Data Systems, Inc.). Twenty-four trials (8 for each delay), with a 2-s inter-trial interval, were completed, with delay intervals randomly interspersed and cue locations randomized over trials. Visual cues were presented randomly at two different locations in each of four quadrants (8 trials) for each delay. Working memory accuracy was computer assessed by use of the hypotenuse of a triangle formed by the actual target location and the vertical and horizontal deviations from the actual target indicated by the participant by use of the light pen. RT was also recorded by computer.

As described previously (Luciana et al., 1992, 1998; Luciana and Collins, 1997), MP drug effects on attentional, arousal, perceptual, and sensorimotor processes involved in a targeted visual search (but not specifically in working memory tasks) were assessed on day 4 by use of (a) a non-mnemonic spatial location task of 16 stimulus trials with no response delay, where accuracy and latency to respond were computer recorded; and (b) a bi-letter cancellation task, where number of omission and commission errors (unmarked target letters and incorrectly marked non-target letters, respectively) were tabulated. Order of these tasks was: non-mnemonic spatial location, working memory task, bi-letter cancellation task. These tasks were given on day 1 and day 4 immediately after all the video clips had been viewed and responded to for affective and motor variables.

### Procedure

Participants were habituated to Labs A and B during two pre-study visits to the labs. Participants completed the 2½ h protocol sometime between noon and 6 p.m. for seven consecutive days. MP and placebo were administered with water in Lab A upon arrival, and tasks and measures occurred over a 1-h period beginning 1 h post-drug ingestion. Participants fasted from midnight prior to each study day, and were on a low monoamine diet for three days prior to and during the study.

## Results

As recommended by others (Anagnostaras and Robinson, 1996; Volkow et al., 1997, 1998; Robinson and Berridge, 2000), magnitude of conditioning was assessed as % change from Association day 1 to Test day 4 on the three dependent variables: motor velocity (finger tapping), positive affect ratings, and visuospatial working memory accuracy. Within the Placebo (PB) and Unpaired (UP) conditions, the high and low extrovert subgroups showed no significant difference on Association day 1 or in % change from day 1 to Test day 4 for any of the five video clips (alpha adjusted for number of analyses, *P* < 0.005). Thus, a 4 (subgroups: PBL, PBH, UPL, UPH) × 5 (video clips) ANOVA with repeated measures on the second factor revealed no significant main effects for subgroups [*F*_(3, 144)_ = 1.45, *P* = 0.36] or video clips [*F*_(4, 144)_ = 1.32, *P* = 0.39] on *motor velocity* on day 1. A 4 (subgroups) × 5 (video clips) ANOVA with repeated measures on the second factor revealed no significant main effects for subgroups [*F*_(3, 144)_ = 1.61, *P* = 0.48] or video clips [*F*_(4, 144)_ = 1.13, *P* = 0.59] on *positive affect* ratings on day 1. Finally, a 4 (subgroups) × 3 (working memory delay intervals) ANOVA with repeated measures on the second factor revealed no significant main effects for subgroups [*F*_(3, 72)_ = 1.39, *P* = 0.38] or delay intervals [*F*_(2, 72)_ = 1.47, *P* = 0.46] on *day 1* for *working memory*.

A 4 (subgroups) × 5 (video clips) ANOVA with repeated measures on the second factor revealed no significant main effects for subgroups [*F*_(3, 144)_ = 1.34, *P* = 0.42] or video clips [*F*_(4, 144)_ = 1.44, *P* = 0.51] on % change from Association day 1 to Test day 4 for *motor velocity*. In addition, a 4 (subgroups) × 5 (video clips) ANOVA with repeated measures on the second factor revealed no significant main effects for subgroups [*F*_(3, 144)_ = 1.21, *P* = 0.54] or video clips [*F*_(4, 144)_ = 1.68, *P* = 0.33] on % change from Association day 1 to Test day 4 for *positive affect* ratings. Finally, a 4 (subgroups) × 3 (working memory delay intervals) ANOVA with repeated measures on the second factor revealed no significant main effects for subgroups [*F*_(3, 72)_ = 1.42, *P* = 0.35] or delay intervals [*F*_(2, 72)_ = 1.39, *P* = 0.42] on % change from Association day 1 to Test day 4 for *working memory*.

Thus, none of the four extraversion subgroups comprising PB and UP experimental conditions showed evidence on motor velocity, positive affect, or working memory of conditioning (i.e., no significant % change from day 1 to day 4 on any measure), nor did they differ significantly from each other on day 1. Therefore, these low and high extraversion subgroups were combined, leaving the larger PB and UP groups (now each with an *N* of 20). The low and high subgroups in the paired condition represent the strong test of differential conditioning, so they were of course not combined.

### Group comparisons of motor velocity and positive affect ratings

**Figure 3:**
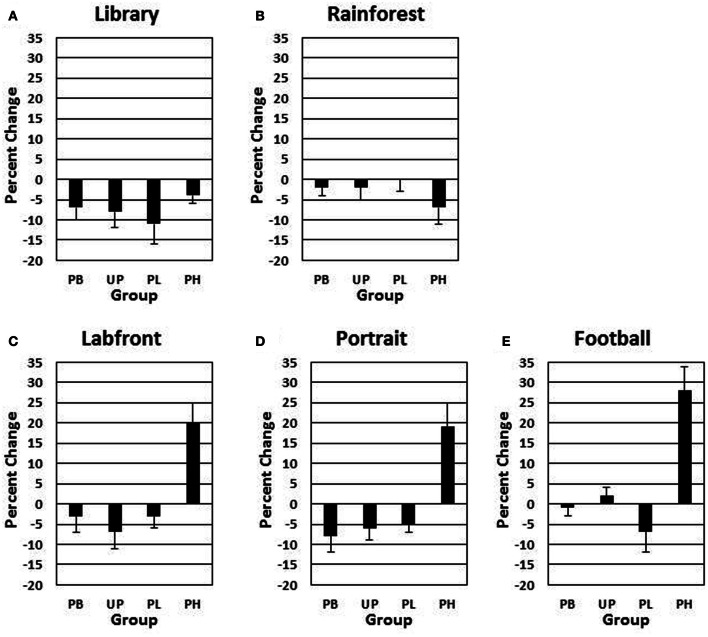
**Conditioned contextual facilitation of motor velocity during the Association phase for four experimental groups**. Shown is the degree of contextual facilitation (% change from Association day 1 to Test day 4) of motor velocity (finger tapping) induced by 5 video clips [*Library* **(A)**, *Rainforest* **(B)**, *Labfront* **(C)**, *Portrait* **(D)**, *Football* **(E)**] in the Association phase. Zero % change indicates no change from day 1 to day 4. PB, placebo; UP, unpaired; PL, paired low extraverts; PH, paired high extraverts.

Alpha adjusted for the number of analyses for the following analyses is *P* < 0.008. A 4 (groups: PB, UP, PL, PH) × 5 (video clips) ANOVA with repeated measures on the second factor revealed no significant main effects for groups [*F*_(3, 272)_ = 1.48, *P* = 0.44] nor for video clips [*F*_(4, 272)_ = 1.51, *P* = 0.51] on day 1 for *motor velocity*. A 4 (groups: PB, UP, PL, PH) × 5 (video clips) ANOVA with repeated measures on the second factor revealed significant main effects for groups [*F*_(3, 272)_ = 19.26, *P* < 0.001; partial eta squared = 0.10] and for video clips [*F*_(4, 272)_ = 15.59, *P* < 0.001; partial eta squared = 0.11] on % change from Association day 1 to Test day 4 for *motor velocity*. The Groups × Video Clips interaction was also significant [*F*_(12, 272)_ = 10.43, *P* < 0.001; partial eta squared = 0.14]. Tukey *post-hoc* comparisons revealed that PH significantly exceeded all of the other three groups in % change for motor velocity on *Labfront, Portrait*, and *Football* video clips (all *P*'s < 0.003), but not on *Library* and *Rainforest* (all P's > 0.30) (Table 2; Figures 3A–E). In addition, none of the other three groups (PB, UP, PL) differed significantly from each other for motor velocity on any of video clips for motor velocity (all *P*'s > 0.30). Indeed, PB, UP, and PL groups generally showed a decrease in % change in motor velocity.

**Table 2:** **Means (SDs) of motor velocity for association and extinction phases**.

**Group**	**PB**	**UP**	**PL**	**PH**
**LIBRARY**				
Day 1	27.12 (3.3)	29.24 (4.1)	28.61 (2.9)	29.03 (3.6)
Day 4	25.31 (3.9)	26.78 (3.7)	25.53 (3.2)	27.81 (3.3)
% change	−7 (3)	−8 (4)	−11 (5)	−4 (6)
**RAINFOREST**				
Day 1	26.23 (2.5)	28.18 (3.5)	29.24 (2.8)	28.93 (3.2)
Day 4	25.68 (3.4)	27.62 (3.3)	29.15 (2.7)	26.78 (3.1)
% change	−2 (2)	−2 (3)	0 (3)	−7 (4)
**LABFRONT**				
Day 1	26.41 (3.8)	28.72 (3.1)	27.33 (3.3)	27.91 (3.5)
Day 4	25.53 (3.5)	26.78 (3.2)	26.47 (3.5)	33.45 (4.2)
% change	−3 (4)	−7 (4)	−3 (3)	20 (5)
Day 5	26.52 (3.2)	27.14 (3.8)	27.11 (3.1)	33.65 (3.8)
% change				21 (8)
Day 7	25.01 (2.4)	25.45 (2.8)	25.95 (3.3)	29.61 (3.2)
% change				6 (5)
**PORTRAIT**				
Day 1	28.03 (4.1)	28.46 (4.1)	28.34 (3.8)	28.51 (3.4)
Day 4	25.71 (3.1)	26.82 (3.3)	27.01 (3.9)	34.02 (4.7)
% change	−8 (4)	−6 (3)	−5 (2)	19 (6)
Day 5	26.13 (3.6)	27.48 (3.4)	27.59 (3.7)	32.86 (4.2)
% change				15 (6)
Day 7	24.91 (4.1)	25.73 (3.3)	27.12 (4.1)	28.17 (3.8)
% change				−1 (4)
**FOOTBALL**				
Day 1	29.32 (3.6)	28.53 (3.2)	29.51 (3.4)	29.26 (3.4)
Day 4	29.11 (3.2)	29.04 (3.4)	25.62 (2.9)	37.45 (4.5)
% change	−1 (2)	2 (4)	−7 (5)	28 (6)
Day5	28.14 (3.7)	28.33 (3.9)	26.04 (3.9)	35.47 (4.4)
% change				21 (6)
Day7	26.17 (3.5)	27.64 (3.3)	25.15 (3.7)	30.14 (3.9)
% change				3 (5)

The association phase represents data for the four groups for Association day 1, Test day 4, and percent (%) change from day 1 to day 4 as a function of stimulus scene. The extinction phase shows data for all groups on days 5 and 7, and % change for only the PH group on days 5 and 7 for the stimulus scenes on which conditioning was observed (Labfront, Portrait, Football). Data are rounded. PB, placebo; UP, unpaired; PL, paired low extraverts; PH, paired high extraverts.

**Figure 4:**
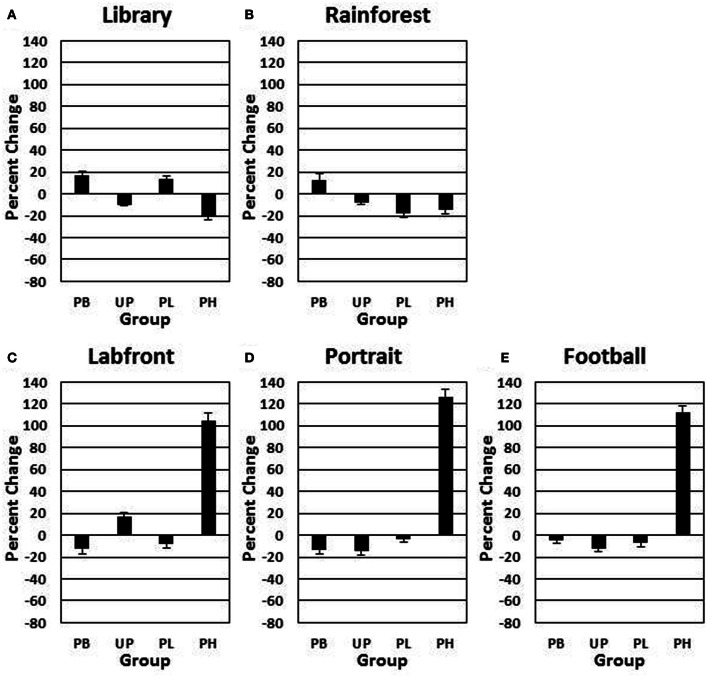
**Conditioned contextual facilitation of positive affect during the Association phase for four experimental groups**. Shown is the degree of contextual facilitation (% change from Association day 1 to Test day 4) of positive affect ratings induced by 5 video clips [*Library* **(A)**, *Rainforest* **(B)**, *Labfront* **(C)**, *Portrait* **(D)**, *Football* **(E)**] in the Association phase. Zero % change indicates no change from day 1 to day 4. PB, placebo; UP, unpaired; PL, paired low extraverts; PH, paired high extraverts.

A 4 (groups: PB, UP, PL, PH) × 5 (video clips) ANOVA with repeated measures on the second factor revealed no significant main effects for groups [*F*_(3, 272)_ = 1.433, *P* = 0.49] nor for video clips [*F*_(4, 272)_ = 1.46, *P* = 0.45] on day 1 for *positive affect* ratings. A 4 (groups: PB, UP, PL, PH) × 5 (video clips) ANOVA with repeated measures on the second factor revealed significant main effects for groups [*F*_(3, 272)_ = 21.37, *P* < 0.001; partial eta squared = 0.17] and for video clips [*F*_(4, 272)_ = 16.92, *P* < 0.001; partial eta squared = 0.15] on % change from Association day 1 to Test day 4 for *positive affect* ratings. The Groups × Video Clips interaction was also significant [*F*_(12, 272)_ = 10.28, *P* < 0.001; partial eta squared = 0.23]. Tukey *post-hoc* comparisons revealed that PH significantly exceeded all of the other three groups in % change for positive affect on *Labfront, Portrait*, and *Football* video clips (all *P*'s < 0.003), but not on *Library* and *Rainforest* (all *P*'s > 0.30) (Table 3; Figures 4A–E). In addition, none of the other three groups (PB, UP, PL) differed significantly from each other on any of video clips for positive affect (all P's > 0.30). Indeed, PB, UP, and PL groups generally showed a decrease in % change in positive affect.

**Table 3:** **Means (SDs) of positive affect ratings for association and extinction phases**.

**Group**	**PB**	**UP**	**PL**	**PH**
**LIBRARY**				
Day 1	1.8 (0.5)	2.1 (0.6)	1.4 (0.4)	1.5 (0.5)
Day 4	2.1 (0.3)	1.9 (0.5)	1.6 (0.5)	1.2 (0.6)
% change	17 (4)	−10 (1)	14 (2)	−20 (4)
**RAINFOREST**				
Day 1	1.5 (0.4)	2.6 (0.7)	2.2 (0.6)	2.6 (0.5)
Day 4	1.7 (0.6)	2.4 (0.6)	1.8 (0.3)	2.2 (0.4)
% change	13 (5)	−8 (2)	−18 (4)	−15 (3)
**LABFRONT**				
Day 1	1.7 (0.8)	1.8 (0.4)	2.4 (0.5)	2.1 (0.6)
Day 4	1.5 (0.7)	2.1 (0.3)	2.2 (0.5)	4.3 (0.7)
% change	−12 (5)	17 (4)	−8 (4)	105 (7)
Day 5	1.4 (0.4)	1.8 (0.5)	1.2 (0.4)	3.8 (0.6)
% change				81 (6)
Day 7	1.2 (0.3)	1.5 (0.6)	1.2 (0.3)	1.5 (0.4)
% change				−29 (5)
**PORTRAIT**				
Day 1	2.4 (0.7)	2.7 (0.7)	2.6 (0.5)	2.7 (0.6)
Day 4	2.1 (0.4)	2.3 (0.8)	2.5 (0.7)	6.1 (0.7)
% change	−13 (4)	−15 (3)	−4 (2)	126 (7)
Day 5	2.2 (0.6)	2.1 (0.6)	2.4 (0.6)	5.7 (0.6)
% change				111 (7)
Day 7	1.8 (0.4)	1.7 (0.5)	1.5 (0.2)	2.1 (0.5)
% change				−22 (4)
**FOOTBALL**				
Day 1	4.3 (0.6)	4.1 (0.6)	4.4 (0.7)	4.3 (0.6)
Day 4	4.1 (0.8)	3.6 (0.5)	4.1 (0.4)	9.1 (0.7)
% change	−5 (2)	−12 (3)	−7 (4)	112 (7)
Day 5	4.2 (0.7)	3.5 (0.4)	3.2 (0.7)	8.8 (0.8)
% change				105 (5)
Day 7	3.8 (0.6)	2.6 (0.5)	3.6 (0.6)	4.3 (0.7)
% change				0 (4)

The association phase represents data for the four groups for Association day 1, Test day 4, and percent (%) change from day 1 to day 4 as a function of stimulus scene. The extinction phase shows data for all groups on days 5 and 7, and % change for only the PH group on days 5 and 7 for the stimulus scenes on which conditioning was observed (Labfront, Portrait, Football). Data are rounded. PB, placebo; UP, unpaired; PL, paired low extraverts; PH, paired high extraverts.

Thus, only PH showed a significant increase in % change from Association day 1 to Test day 4 in both motor velocity and positive affect to the three video clips that were either paired with MP and Lab A context (*Labfront, Portrait*) or had high inherent incentive value (*Football*). PH did not evidence increases in % change for video clips that were not paired with MP or Lab A context (*Library*) or that had low inherent incentive value (*Rainforest*). The % change increase in motor velocity by PH was substantial, ranging from increases of 19–28%, being greatest for *Football*. The % change increase in positive affect ratings by PH was particularly substantial, ranging from increases of 105–126%, being greatest for *Portrait* [note that although the female Portrait may have been more rewarding to the male participants, this analysis was on the change from day 1 to day 4, and hence represents a conditioning effect only]. For PH, within-subject increases in % change in motor x affect variables correlated (Pearson product-moment) significantly for *Labfront* (*r* = 0.49, *P* < 0.05), Portrait (*r* = 0.52, *P* < 0.05), and *Football* (*r* = 0.50, *P* < 0.05), indicating a joint conditioned contextual facilitation across two different DA-modulated response systems within participants.

### Group comparisons of visuospatial working memory

**Figure 5:**
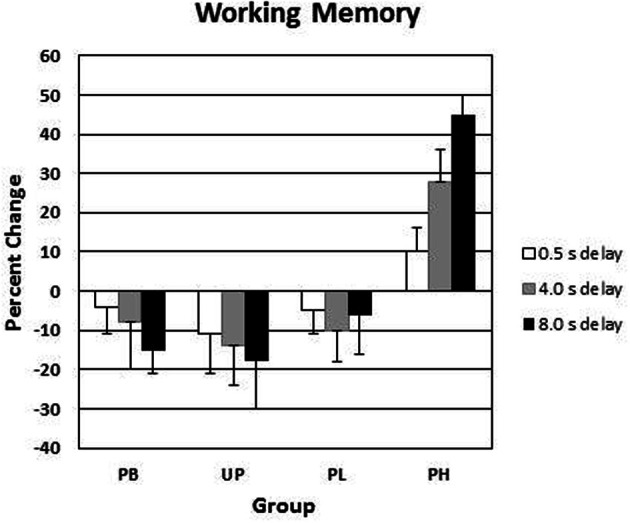
**Conditioned contextual facilitation of visuospatial working memory during the Association phase for four experimental groups**. Shown is the degree of contextual facilitation (% change from Association day 1 to Test day 4) of visuospatial working memory induced by the general context of Lab A in the Association phase. PB, placebo; UP, unpaired; PL, paired low extraverts; PH, paired high extraverts.

Alpha was adjusted to number of analyses at *P* < 0.03. A 4 (groups: PB, UP, PL, PH) × 3 (delay intervals) ANOVA with repeated measures on the second factor revealed no significant main effects for groups [*F*_(3, 136)_ = 1.53, *P* < 0.39] nor for delay intervals [*F*_(2, 136)_ = 1.49, *P* < 0.34] on day 1 for *visuospatial working memory accuracy*. A 4 (groups: PB, UP, PL, PH) × 3 (delay intervals) ANOVA with repeated measures on the second factor revealed significant main effects for groups [*F*_(3, 136)_ = 18.45, *P* < 0.001; partial eta squared = 0.18] and for delay intervals [*F*_(2, 136)_ = 21.72, *P* < 0.001; partial eta squared = 0.23] on % change from Association day 1 to Test day 4 for *visuospatial working memory accuracy*. The Groups × Delay interaction was also significant [*F*_(6, 136)_ = 13.13, *P* < 0.001; partial eta squared = 0.31] (Table 4; Figure 5). Tukey *post-hoc* comparisons revealed that the four groups did not differ in % change from day 1 to day 4 in working memory accuracy for the delay interval of 0.5 s (all *P*'s>0.30). However, PH significantly exceeded all of the other three groups in % change for working memory accuracy at delay intervals of 4.0 s and 8.0 s (all *P*'s<0.003). None of the other three groups (PB, UP, PL) differed significantly from each other at any of the delay intervals (all *P*'s >0.30). Indeed, PB, UP, and PL groups showed decreases in % change in working memory accuracy at all delay intervals. Finally, PH showed a significant increase in % change from delay interval 0.5 s to 4.0 s (*P* < 0.003), as well as a significant increase in % change from delay interval 4.0 s to 8.0 s (*P* < 0.003) (see Table 4 and Figure 5). The % change increases for PH were substantial, ranging from +29% at delay 4.0 s to +47% at delay 8.0 s, which is in accord with the demands on DA functioning in dorsolateral prefrontal cortex during increasingly long working memory delay periods (Luciana et al., 1992, 1998; Luciana and Collins, 1997).

**Table 4:** **Means (SDs) for % change in visuospatial working memory in the association phase**.

**Delay interval**	**PB**	**UP**	**PL**	**PH**
0.0 s	−4 (3)	−8 (11)	−2 (5)	−3 (4)
0.5 s	−5 (8)	−7 (9)	−6 (6)	11 (6)
4.0 s	−9 (12)	−12 (10)	−12 (8)	29 (8)
8.0 s	−14 (7)	−15 (14)	−7 (11)	47 (6)

PB, placebo; UP, unpaired; PL, paired low extraverts; PH, paired high extraverts.

For PH participants, the % change increase at 8.0 s delay correlated significantly with the % change increase in motor velocity (*r* = 0.49, *P* < 0.05) and positive affect (*r* = 0.57, *P* < 0.05) to the *Football* video clip, again indicating a joint conditioned contextual within-subject facilitation across three different DA-modulated response systems within participants. [Affective responses to the *Football* clip were used here to correlate with the other dependent variables, because it had the strongest affective induction of positive affect].

Finally, MP drug effects on attentional, arousal, perceptual, and sensorimotor processes involved in a targeted visual search (but not specifically in working memory) were assessed by use of a non-mnemonic spatial location task of 16 stimulus trials with no response delay (0.0 s) on day 4, where accuracy was computer recorded. Adjusted alpha was *P* < 0.007. There was no significant main effect for One-Way ANOVA's comparing accuracy [*F*_(3, 64)_ = 1.23, *P* = 0.45] or RT [*F*_(3, 64)_ = 1.51, *P* = 0.48] of the four groups at a delay of 0.0 s. In addition, a bi-letter cancellation task was also used to assess MP drug effects on attentional, arousal, perceptual, and sensorimotor processes on day 4, where number of omission + commission errors (unmarked target letters + incorrectly marked non-target letters, respectively) were tabulated. There were no significant main effects for the four groups in a One-Way ANOVA in bi-letter accuracy scores [*F*_(3, 64)_ = 1.43, *P* = 0.42]. Taken together, these findings indicate that MP effects on attentional, arousal, perceptual, and sensorimotor processes do not account for group differences in the working memory results.

### Motor velocity and positive affect in the extinction phase

**Figure 6:**
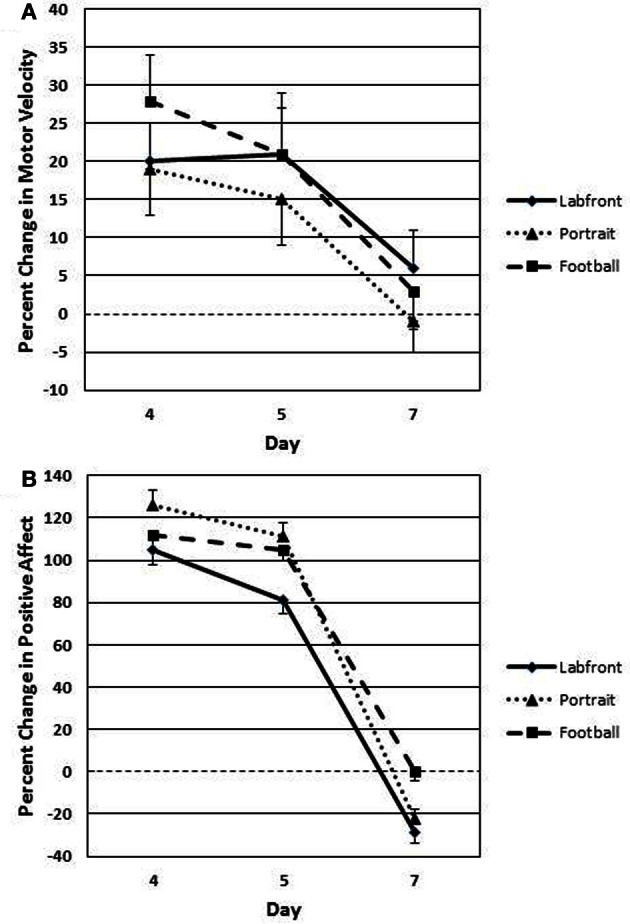
**Extinction (placebo during days 5, 6, and 7) of conditioned contextual facilitation of motor velocity (A) and positive affect (B) to successfully conditioned video clips (*Labfront, Portrait*, and *Football*) in PH participants (who were the only participants to condition)**. Degree of extinction of conditioned contextual facilitation is indexed as % change (change from day 1) in responding on Test day 4, day 5, and day 7. Responding on day 5 is a strong index of conditioning in that facilitated responding (degree of similarity to facilitated responding on Test day 4) occurs only to context, because the unconditioned effects of methylphenidate are absent. PH, paired high extraverts.

Extinction-phase data represent % change in motor velocity and positive affect from day 1 to each of days 4, 5, and 7 (% change in days 1 to 4 is used as the conditioning baseline for assessing extinction effects). Because only PH demonstrated significant conditioning (all other groups showed a level line across days 4–7; Tables 2, 3), only the PH Extinction data are analyzed for the three video clips that evidenced conditioning: *Labfront, Portrait*, and *Football* (Table 4; Figures 6A,B). Alpha was adjusted for number of analyses at *P* < 0.13. A 3 (video clips) × 3 (days 4, 5, 7) ANOVA with repeated measures on both factors revealed a significant main effect for days [*F*_(2, 84)_ = 14.37, *P* < 0.001; partial eta squared = 0.15], but no significant main effect for video clips [*F*_(2, 84)_ = 1.92, *P* = 0.43], on % change in *motor velocity* (Figure 6A) from Association day 1 to day 4, 5, and 7. Tukey *post-hoc* tests showed that % change on Test day 4 vs. first extinction day 5 was not significant for any of the three video clips (all *P*'s > 0.30), indicating that conditioned contextual facilitation occurred on day 5 in the absence of unconditioned MP drug effects. Comparison of % change on day 5 vs. day 7 showed that day 5 significantly exceeded day 7 for all three video clips (all *P*'s < 0.003). As seen in Figure 6A, by day 7 motor responding was at or near the level of day 1 (indicated by the 0% change dashed line) on all three video clips.

A 3 (video clips) × 3 (days 4, 5, 7) ANOVA with repeated measures on both factors revealed a significant main effect for days [*F*_(2, 84)_ = 19.42, *P* < 0.001; partial eta squared = 0.28], but no significant main effect for video clips [*F*_(2, 84)_ = 1.62, *P* = 0.38], on % change in *positive affect* (Figure 6B) from Association day 1 to day 4, 5, and 7. Tukey *post-hoc* tests showed that % change on day 4 vs. day 5 was not significant for any of the three video clips (all *P*'s > 0.30), indicating that conditioned contextual facilitation occurred on day 5 in the absence of unconditioned MP drug effects. Comparison of % change on day 5 vs. day 7 showed that day 5 significantly exceeded day 7 for all three video clips (all *P*'s < 0.003). As seen in Figure 6B, by day 7 positive affect ratings were at or below the level of day 1 (indicated by the 0 % change dashed line) on all three video clips.

## Discussion

The current findings suggest that extraversion is positively related to brain processes that associate contexts with reward. The robustness of this conclusion is indicated by five findings:

- There was a significant acquired contextual facilitation of responding in PH but little-to-none in PL across Association day 1 to Test day 4 in motor velocity, positive affect, and working memory. In fact, PL generally showed decreased levels of responding from day 1 to day 4 on all measures. In contrast, enhanced responding by PH on Test day 4 relative to Association day 1 was substantial, ranging across variables from increases of 19–21% for motor velocity, 105–126% for positive affect, and 29 and 47% for working memory in delays of 4.0 s and 8.0 s, respectively. No such facilitation was found in PH with stimuli that had not been associated with MP (i.e., *Library* and *Rainforest*) or had no inherent incentive value (Rainforest).
- *Breadth* of acquired contextual facilitation across motor, affective, and cognitive processes occurred in PH but not PL. Moreover, conditioned facilitation in PH was also found equally for visual stimuli that differ in their ease and strength of conditioning (Holland, 1992; Graybiel, 1998) [implicit, contextual stimuli (*Labfront*) vs. explicit, discrete stimuli (*Portrait*)], and that are likely processed along different brain pathways [i.e., ventral (*Portrait*) and dorsal (*Labfront*) visual streams]. Thus, broad conditioned contextual facilitation was observed across different domains (motor, affective, and cognitive) and for different types of stimuli (general context and a discrete object stimulus) for PH participants.
- There were significant *correlations within participants* across combinations of all three domains (motor, affective, cognitive), ranging from 0.46 to 0.52.
- There was robust conditioned contextual facilitation by PH on the first day of Extinction (day 5), despite the absence of unconditioned effects of MP.
- Non-specific, general contextual stimuli (i.e., Lab A) elicited enhanced facilitation of responding on day 4 relative to day 1 in PH participants to visual stimuli that are naturally of high incentive salience (*Football*), but not to stimuli of little incentive salience (*Rainforest*) (Jodogne et al., 1994; Schultz et al., 1997; Robinson and Berridge, 2000). Therefore, according to the rationale described in the Materials and Methods section, one may conclude that the enhanced response to *Football* on day 4 was dependent on contextual conditioning in PH participants only (Robinson and Berridge, 2000).

Thus, high extraverts that had context paired with MP in Lab A during the Association phase of the study (i.e., PH) manifested broad conditioned contextual facilitation across motor, affective, and cognitive processes, where the three processes correlated in magnitude of facilitation within participants, and which persisted into the first day of Extinction when no unconditioned effects of MP were present. These conditioned effects were not observed in high or low extraverts who had no exposure to MP in Lab A (i.e., PB and UP), or who had been exposed to MP but in a different lab context (i.e., UP in Lab B). Indeed, PB and UP groups generally showed a moderate loss of contextual facilitation on Test day 4 relative to Association day 1, apparently due to having found repeated presentation of the Lab A context to be absent of incentive value without MP exposure.

Most importantly, low extraverts exposed to MP in Lab A (i.e., PL) apparently experienced little or no rewarding effects from the MP dose used in this study, since they manifested no significant conditioned contextual facilitation on Test day 4 relative to Association day 1. This suggests that PH participants are more sensitive than PL participants to the MP-induced reward generated by the dose used here. This would support the notion that extraversion is characterized by individual differences in reactivity to reward or incentive stimuli, and that these differences have implications for contextual conditioning (Depue et al., 1994; Gray, 1994; Depue and Collins, 1999).

Several lines of evidence suggest that DA modulation contributes to the relation between extraversion and the magnitude of conditioned contextual facilitation of responding. First, DA functioning in the NAc in animals is strongly correlated with (a) the acquisition of reward-induced conditioned contextual responding (Hooks et al., 1992; Cabib, 1993; Jodogne et al., 1994; Wassum et al., 2011), (b) the magnitude of incentive attributed to context (Hooks et al., 1992; Cabib, 1993; Jodogne et al., 1994; Robinson and Berridge, 2000), and (c) the efficacy of drug-associated cues to markedly enhance DA release and gene expression in the NAc (Berke and Hyman, 2000; Everitt et al., 2001). Second, as reviewed above, MP is a potent DA agonist and inducer of feelings of reward in humans. It was the pairing of MP with context in our study that was critical to demonstrating contextual facilitation in PH participants in that equivalently high extraverts in conditions that did not pair MP with context (i.e., PB and UP participants) did not acquire such conditioned facilitation. Third, the presence of conditioned facilitation in PH participants on the first day of Extinction (where no unconditioned MP effects were present) is also consistent with cue-induced NAc DA activity (Ranaldi et al., 1999; Devilbiss and Berridge, 2008). Fourth, as discussed above, the dependence of facilitation of motor velocity, positive affect, and visuospatial working memory processes on VTA DA projections to the NAc and dorsolateral prefrontal cortex, respectively, is well established in animals and humans (Luciana et al., 1992, 1998; Luciana and Collins, 1997; Depue and Collins, 1999; Devilbiss and Berridge, 2008; McNab et al., 2009; Aart et al., 2011). Fifth, the increasing efficacy of contextual facilitation of working memory with longer response delays found here, when demands on DA facilitation are increasing, is also consistent with a role for DA (Luciana et al., 1992, 1998; Luciana and Collins, 1997). And sixth, that only PH but not PL participants acquired a context-incentive reward association may reflect the positive relation between DA functioning and extraversion reviewed above.

VTA DA neural subgroups positioned more laterally in midbrain project to the NAc, where DA release enhances incentive facilitation of locomotor activity and positive affect (Depue and Collins, 1999; Olson et al., 2005; Fields et al., 2007). In contrast, more medially located VTA DA neural subgroups project to cortical regions, such as the dorsolateral prefrontal cortex, and facilitate working memory processes (Goldman-Rakic, 1987; Luciana et al., 1992, 1998; Fields et al., 2007). The fact that incentive motivational processes reflected by motor and affective variables, as well as cognitive processes indexed by visuospatial working memory, similarly evidenced conditioned contextual facilitation, and that these three variables correlated in % change with each other within participants, suggests that afferents from corticolimbic regions carrying contextual information to the VTA have broad excitatory effects across distinct VTA DA nuclear subgroups (Oades and Halliday, 1987; Taber et al., 1995; Luciana et al., 1998; Groenewegen et al., 1999b; Berke and Hyman, 2000; Carr and Sesack, 2000). Thus, contexts that have been associated with reward appear to facilitate not only incentive motivational processes that activate approach to reward (Berke and Hyman, 2000; Hyman and Malenka, 2001), but also cognitive processes that mediate behavioral strategies and outcome expectancies that guide goal-oriented decisions and behaviors (Everitt et al., 2001; Hyman and Malenka, 2001). This perspective suggests that extraversion involves both affective and cognitive components in engaging with rewarding goals (Gray and Braver, 2002; Depue and Fu, 2012).

The conditioned contextual effects found in PH are specific to the trait of extraversion. This is because we used selection criteria that limited our participants to the middle six deciles on the two major higher-order traits of neuroticism and constraint (impulsivity). While this selection method helps to assure specificity of results to extraversion, it also creates study participants that do not represent the full range of combinations of extraversion with other higher-order traits. Such combinations (e.g., high extraversion and low constraint) may modify conditioning effects (Depue and Fu, 2012). Future studies will need to assess the effects of interactions of traits on the conditioning process.

**Figure 7:**
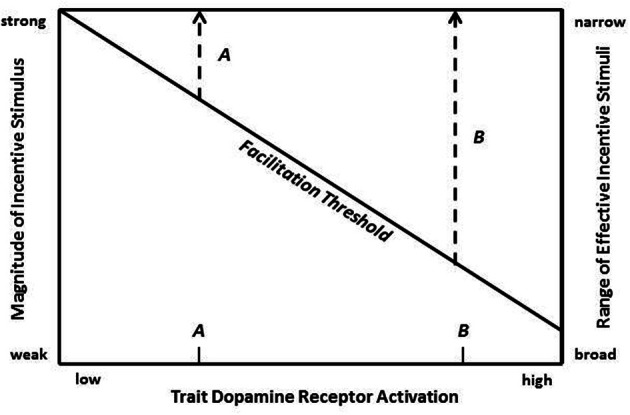
**A minimum threshold for facilitation of feelings of reward and extraverted behavior is illustrated as a trade-off function between incentive stimulus magnitude (left vertical axis) and dopamine (DA) postsynaptic receptor activation (horizontal axis)**. Range of effective (facilitating) incentive stimuli is illustrated on the right vertical axis as a function of level of DA activation. Two hypothetical individuals with low and high trait DA postsynaptic receptor activation (demarcated on the horizontal axis as A and B, respectively) are shown to have narrow (A) and broad (B) ranges of effective incentive stimuli, respectively.

At a broader level, the current findings shed further light on the nature of extraversion. Two points are worth emphasizing about extraversion. First, as much research in genetics, pharmacology, psychology, and neuroscience now suggests, a major contributor to variation in extraverted behavior is individual differences in the functional properties of the VTA DA-NAc/cortical pathways. Second, variation in DA functioning is manifested by the eliciting effects of environmental incentive stimuli, which as our study suggests can be conditioned incentives as well. Therefore, as shown in Figure 7, the expression of extraverted behavior can be illustrated by a threshold model that represents a central nervous system weighting of the external and internal factors that contribute to initiation of behavior (Stricker and Zigmond, 1986; White, 1986; Depue and Collins, 1999). In the case of extraversion, the threshold would be weighted most strongly by the joint function of two main variables: (i) the magnitude of incentive stimuli, which ultimately is mainly a function of the magnitude of reward induced by an unconditioned or conditioned incentive stimulus, and (ii) level of DA postsynaptic receptor activation. The interaction of these two variables creates a trade-off function in Figure 7, where pairs of values (of incentive stimulus magnitude and DA activation) specify a diagonal representing the minimum threshold value for activation of incentive reward processes that manifest as extraverted behavior. Because the two input variables are interactive, independent variation in either one not only modifies the probability of behavior, but it also simultaneously modifies the value of the other variable that is required to reach a minimum threshold of reward and extraverted behavior.

A threshold model allows behavioral predictions that have implications for conceptualizing the nature of extraversion. A *trait* dimension of DA postsynaptic receptor activation is represented on the horizontal axis of Figure 7, where two individuals with divergent trait levels are demarcated: *A* (low trait level) and *B* (high trait level). These two divergent individuals may be used to illustrate the effects of trait differences in DA receptor activation on both acquisition and maintenance of extraverted behavior.

First, as Figure 7 indicates, for any given incentive stimulus, the degree of DA response will on average be larger in individual *B* vs. *A*. Because the degree of DA activity is correlated with the magnitude of *positive affect* that is naturally elicited by incentive stimuli [e.g., increased enthusiasm, activity, desire, wanting, optimism], this positive emotional experience is also predicted to be more enhanced in *B* vs. *A*.

*Second*, trait differences in incentive activation may have marked effects on the *range* of effective (i.e., reward- and behavior-inducing) incentive stimuli. This is illustrated in Figure 7, where the right vertical axis represents the range of effective affiliative stimuli. Increasing trait levels of DA activation (horizontal axis) are associated with an increasing efficacy of weaker incentive stimuli and, thus, with an increasing range of effective incentive stimuli. In Figure 7 individuals *A* and *B* have a narrow vs broad range, respectively. Significantly, the broader range for individual *B* suggests that on average *B* will experience more *frequent* elicitation of positive emotional experiences associated with reward.

Third, if individual B experiences more frequent and more enhanced reward to incentive sitmuli, animal research suggests that this experience is associated with the quantity of DA release in the NAc and with a graded increase in the frequency and duration of VTA DA neuronal activity (White, 1986; Nishino et al., 1987; Blackburn et al., 1989; Schultz et al., 1995). Thus, variation in DA activation by incentive stimuli may not only influence the level of experienced reward, but also may lead to variation in the strength of DA-facilitated associative processes that link neutral stimuli with reward (Phillips et al., 2003; Simmons and Neill, 2009; Wassum et al., 2011). *The outcome of these interactions may be the acquisition of a more elaborate associative network linking reward to incentive stimuli in individual B*. The findings of the current study support such a proposition.

Finally, the *maintenance* of individual differences in extraversion may relate to the very factors that promote variation in the acquisition of conditioned incentive stimuli. The latter would be expected to result in variation in the strength and breadth of the encoded memory *network* of conditioned positive incentives (i.e., a contextual ensemble) that represents the general context and specific features associated with subsequent reward. Such differences in reward-encoding of memory representations of salient contexts could have marked effects on the maintenance of extraverted behavior through the operation of cognitive processes of working memory integrated in prefrontal cortical regions. In prefrontal regions, symbolic central representations of the salient context associated with reward can be held on-line as a means of (a) “reliving” and predicting the expected reward from engagement with a salient context, and (b) guiding motivated approach to the goal (Goldman-Rakic, 1987; Waterhouse et al., 1996; Damasio, 1999; Rolls, 2000). Thus, individuals *A* and *B* may develop *differences in their capacity to facilitate over time subjective reward and extraverted behavior due to differentially encoded central representations of salient contexts and their expected outcome (most likely held in mOFC* (Depue and Collins, 1999). Put differently, individual differences in extraversion may be *maintained* by activation of differentially encoded central representations of incentive contexts that predict reward. The implications of the current study are that, in high extraverts, who are predicted to have a lower threshold of behavioral facilitation, this process will involve: (i) more *frequent* activation of incentive; (ii) by a *broader* network of conditioned contexts that; (iii) elicit more strongly encoded central representations of related rewarding events and their expected outcomes.

### Conflict of interest statement

The authors declare that the research was conducted in the absence of any commercial or financial relationships that could be construed as a potential conflict of interest.
